# Comparison of EMC and CM methods for orienting diffraction images in single-particle imaging experiments

**DOI:** 10.1107/S205225252100868X

**Published:** 2021-10-07

**Authors:** Miklós Tegze, Gábor Bortel

**Affiliations:** a Institute for Solid State Physics and Optics, Wigner Research Centre for Physics, Konkoly Thege Miklós út 29-33, Budapest, H-1121, Hungary

**Keywords:** single-particle imaging, orientation methods, diffraction patterns, correlation-maximization algorithms, expansion-maximization-compression algorithms

## Abstract

In single-particle imaging (SPI) experiments, diffraction patterns of identical particles are recorded. The particles are injected into the X-ray free-electron laser (XFEL) beam in random orientations. The crucial step of the data processing of SPI is finding the orientations of the recorded diffraction patterns in reciprocal space and reconstructing the 3D intensity distribution. Here, two orientation methods are compared: the expansion maximization compression (EMC) algorithm and the correlation maximization (CM) algorithm. To investigate the efficiency, reliability and accuracy of the methods at various XFEL pulse fluences, simulated diffraction patterns of biological molecules are used.

## Abstract 2

Two orientation methods are compared: the expansion maximization compression (EMC) algorithm and the correlation maximization (CM) algorithm. To investigate the efficiency, reliability and accuracy of the methods at various X-ray free-electron-laser pulse fluences, simulated diffraction patterns of biological molecules are used.

## 1. Introduction   

The short and intense pulses of X-ray free-electron lasers (XFELs) make diffraction experiments on single particles possible (Neutze *et al.*, 2000 ▸; Huldt *et al.*, 2003 ▸). In a single-particle imaging (SPI) experiment, identical particles are injected into the X-ray beam with random orientations and diffraction patterns can be recorded in a 2D detector before the particle is destroyed by radiation damage. Individual diffraction patterns of small particles or molecules are noisy and contain insufficient information to solve the structure of the particle. Therefore, to assemble a single set of consistent diffraction data, thousands or millions of diffraction patterns must be recorded (Poudyal *et al.*, 2020 ▸).

To solve the structure of the particle starting from the raw detector images, three major steps are necessary:

(i) Converting the raw detector data to diffraction patterns of single particles. The XFEL pulse can hit no or multiple particles, so first, detector images with scattering data on single particles are selected. Then, detector noise is removed and the detector data is converted into photon counts. Background noise, recorded in no-hit images, should be also removed.

(ii) Finding the relative orientations of the diffraction patterns in reciprocal space and assembling a consistent 3D diffraction intensity distribution. The diffraction patterns are very noisy and contain only a low number of photons. In most cases, the particles are randomly oriented and no independent orientation data are available.

(iii) Solving the phase problem to obtain the electron density of the particle. Unlike in conventional crystallography, finding the unknown phases is facilitated by oversampling the reciprocal space (Miao *et al.*, 1999 ▸).

In this article, we concentrate on the second (orientation step) although its effect on the third (phase-recovery step) is also addressed. The diffraction image measured by a 2D detector corresponds to a spherical section (part of the surface of the Ewald sphere) in reciprocal space. The center of the section is always at the origin (**q** = 0), but the orientation of the spherical section (fixed to the orientation of the particle) is unknown. For a small particle, the measured diffraction patterns are extremely noisy. Most of the pixels of a megapixel pattern are empty, only a few hundred or about a thousand pixels have one or a few photon counts.

Several methods have been developed to find the unknown orientations of the diffraction patterns. Many of these methods are related to orientation methods developed for cryo-EM (van Heel, 1987 ▸; van Heel *et al.*, 2000 ▸; Penczek *et al.*, 1994 ▸; Radermacher, 1994 ▸; Sigworth, 1998 ▸; Sigworth *et al.*, 2010 ▸). Cryo-EM records the absorption of the particles that is proportional to the 2D projection of the electron density. According to the central-slice theorem, the Fourier transform of the measured projections are central slices (planes through the origin) in the 3D reciprocal space. Orienting these central slices is a task similar to the SPI orientation problem. However, there are important differences. In SPI, the diffraction images are not flat, they are parts of the Ewald sphere. On the other hand, in cryo-EM, the common center of the real-space projections is not known. Therefore, not only the three orientation angles but also two additional shift parameters should be found for each image. Because of these differences, while the SPI orientation methods may use approaches similar to those of cryo-EM, the details of the algorithms can be quite different.

One group of methods to orient the diffraction patterns in SPI relies on the information in the common intersection curves of the patterns (Huldt *et al.*, 2003 ▸; Shneerson *et al.*, 2008 ▸; Bortel & Tegze, 2011 ▸; Yefanov & Vartanyants, 2013 ▸; Zhou *et al.*, 2014 ▸). Other methods find the possible orientations of the patterns by comparing them with a 3D intensity model updated by every iteration (Loh & Elser, 2009 ▸; Tegze & Bortel, 2012 ▸; Flamant *et al.*, 2016 ▸; Nakano *et al.*, 2017 ▸, 2018 ▸). Another group of methods uses the manifold embedding technique to find the similarities between diffraction patterns and order them in the orientation space (Fung *et al.*, 2009 ▸; Giannakis *et al.*, 2012 ▸; Kassemeyer *et al.*, 2013 ▸; Winter *et al.*, 2016 ▸). Donatelli *et al.* (2017) ▸ proposed a method to find the orientations and the phases simultaneously. Correlation-based approaches aim to solve the structure without determining the relative orientations of the individual diffraction patterns (Kam, 1977 ▸; Saldin *et al.*, 2009 ▸, 2011 ▸; Elser, 2011 ▸; Donatelli *et al.*, 2015 ▸; von Ardenne *et al.*, 2018 ▸).

In this study, we consider two methods for orientating the measured diffraction patterns and reconstructing the 3D scattering intensity distribution: the expansion maximization compression (EMC) algorithm (Loh & Elser, 2009 ▸; Loh *et al.*, 2010 ▸) and the correlation maximization (CM) algorithm (Tegze & Bortel, 2012 ▸, 2013 ▸, 2016 ▸, 2018 ▸). We compare the efficiency, reliability and accuracy of these methods using simulated measurements on biological molecules at various XFEL pulse fluences. We study the performance of the methods first in a simplified 1D orientation problem. For the full 3D orientation problem, a more detailed comparison of the methods is presented. We compare the results with two references: the ideal noiseless model intensity distribution and the one reconstructed from the noisy diffraction patterns using their true orientations. Finally, we reconstruct the electron densities by solving the phase problem and compare them with the original atomic structure.

## 2. The EMC and CM algorithms   

**Figure 1:**
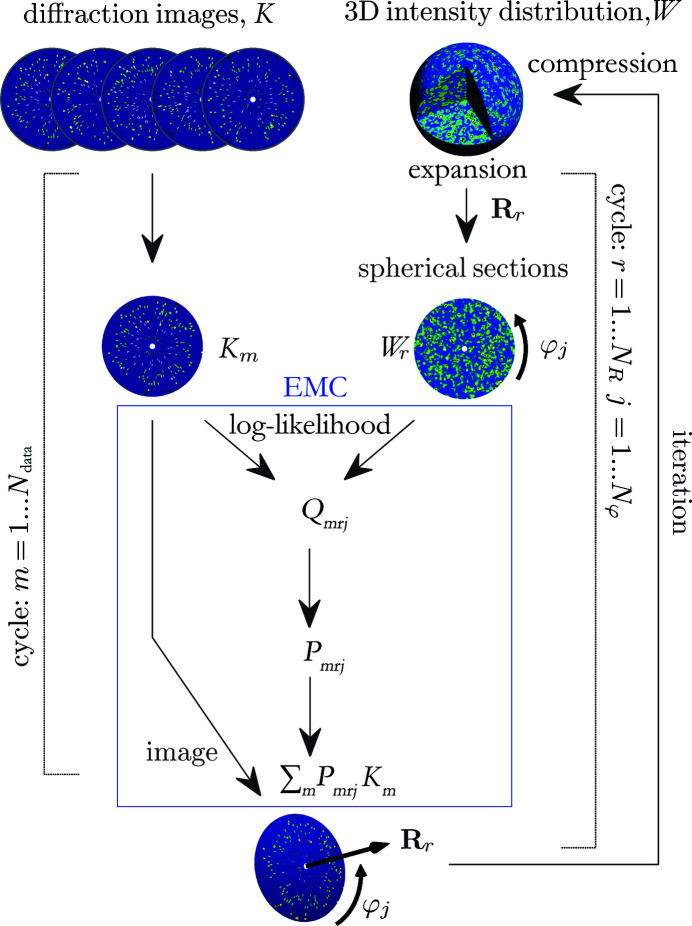
A flowchart of the modified EMC method. The expectation-maximization step is inside the blue box.

The EMC algorithm was developed by Loh & Elser (2009 ▸) and is based on Bayesian information theory. Similar algorithms have been applied in cryo-EM (Sigworth, 1998 ▸; Sigworth *et al.*, 2010 ▸). The name of the EMC algorithm is from the initials of its main steps: expansion, expectation maximization and compression. In the expansion step, a 3D intensity model is expanded into a set of 2D spherical slices corresponding to all possible orientations of the measured diffraction patterns. The central part of the method is expectation maximization: calculating the probabilities that an experimental image was measured in a given orientation with the actual 3D intensity model as condition. Then, in the compression step, these probabilities are used as weights to construct an improved 3D model (Fig. 1 ▸). Starting from a random distribution, after several iterations, the 3D intensity distribution converges to a solution and the probabilities will peak around the true orientations. A detailed description of the algorithm is found in Loh & Elser (2009 ▸). The algorithm is implemented in the computer program package *Dragonfly* (Ayyer *et al.*, 2016 ▸). However, in this study, we did not use this package. To speed up the calculation, we slightly modified the EMC algorithm and wrote a more efficient computer code. The most time-consuming step of the EMC algorithm is the calculation of the probabilities of all possible orientations for all measured diffraction patterns. We introduced polar coordinates and used fast Fourier transform (FFT) and the correlation theorem (Weisstein, 2021 ▸) to increase computational efficiency. Details of the modification are described in the Appendix[App appa]. This modification of the EMC algorithm not only decreases the computing time but also improves its time scaling with the complexity of the particle from *R* ^6^ to *R* ^5^ (for the definition of *R* see Table 1 ▸).

**Figure 2:**
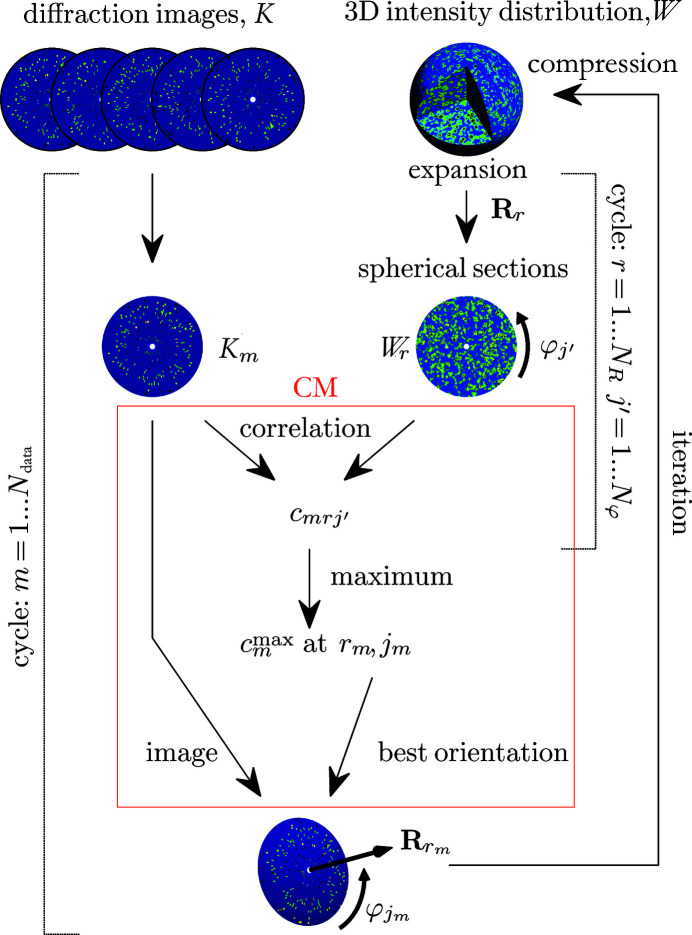
A flowchart of the CM method. The CM step (replacing the expectation-maximization step of the EMC method) is inside the red box.

The CM algorithm is a simplified version of the EMC algorithm, developed by the present authors (Tegze & Bortel, 2012 ▸). A similar approach was suggested earlier for cryo-EM (Penczek *et al.*, 1994 ▸). In the CM method, the time-consuming expectation-maximization step is replaced by a search for the orientation with the highest correlation (Fig. 2 ▸). This is practically equivalent to setting the largest weight among the possible orientations of a diffraction pattern to one and all the others to zero in the EMC method.

Since only the central parts of the algorithms are different (see Figs. 1 ▸ and 2 ▸), both algorithms were implemented in a common framework. The computer program was written in Matlab with parts in C and CUDA for parallel computing on graphics processing units. The calculations were executed on a single workstation (2 × Intel Xeon Gold 6146 computing processor unit) reinforced with a group of graphics processors (8 × Nvidia Geforce RTX 2080 Ti).

## 3. Simulation of diffraction patterns   

The success of the orientation process depends on the experimental parameters: XFEL wavelength, pulse length and fluence, size and composition of the particle, detector efficiency, noise, size, distance and pixel size, number of successfully recorded images of a single particle, background noise, *etc*. Since we wanted to study how the orientation methods perform at different XFEL pulse fluences, all other parameters were fixed at realistic values (listed in Table 1 ▸). Although low-resolution experimental single-particle images are available (Reddy *et al.*, 2017 ▸; Ayyer *et al.*, 2019 ▸), we decided to use simulated scattering patterns at near-atomic resolution. Two test molecules were used: lysozyme, a small protein molecule which has a well known structure (Protein Data Bank; PDB entry 3lzt; Walsh *et al.*, 1998 ▸), and the much larger multiprotein-complex RNA polymerase II (PDB entry 1wcm; Armache *et al.*, 2005 ▸). Many thousands (20 000 for lysozyme and 100 000 for RNA polymerase II) of diffraction patterns of the molecules in random orientations were calculated [as described in Tegze & Bortel (2012 ▸)]. Parameters of the simulations are shown in Table 1 ▸. Poisson noise was introduced for several values of XFEL pulse fluence *I*_0_ in the range of 5 × 10^26^–10^28^ photons m^−2^ for lysozyme and 10^24^–10^26^ photons m^−2^ for RNA polymerase II. For simplicity, the diffraction patterns were generated on the polar grid and detector or background noise (which would have to be removed before further processing) was not added. The XFEL pulse was supposed to be short enough that atomic displacements due to electrostatic forces (Jurek *et al.*, 2004 ▸) are negligible before the pulse ends.

## 4. Comparison of the efficiency and accuracy of the orientation algorithms   

### 4.1. Orientation problem in one dimension   

It is always useful to study a difficult problem first in lower dimensions. The simplest 1D version of the orientation problem of SPI is the following: random sample rotation is allowed only about the incident XFEL beam direction. The scattered radiation is ‘measured’ only in a circle with a fixed scattering angle. For further simplicity, only rotations of integer multiple Δφ are allowed, where Δφ is the azimuthal size of the detector pixel. For this study, simulated 1D scattering patterns of the lysozyme molecule were used. Scattering intensities of *W*_0_(φ_*m*_), where φ_*m*_ = *m*Δφ, were calculated in steps of 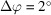 for X-ray wavelength λ = 1 Å at scattering angle 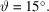 After applying random rotations (their angle saved for later use) about the incident X-ray beam direction, the intensities were multiplied by the solid angle of the detector pixel (for compatibility with the 3D orientation problem, 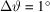 was chosen) and Poisson noise was introduced. We generated *N*_data_ = 20 000 randomly rotated ‘measurements’, 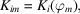 where 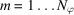 and 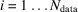 , for each incident X-ray fluence value. To test the accuracy of the results of the orientation methods, an ideal intensity distribution *W*_T_(φ_*m*_) was calculated by averaging all ‘measured’ noisy intensities rotated back by the true rotation angles saved earlier.

**Figure 3:**
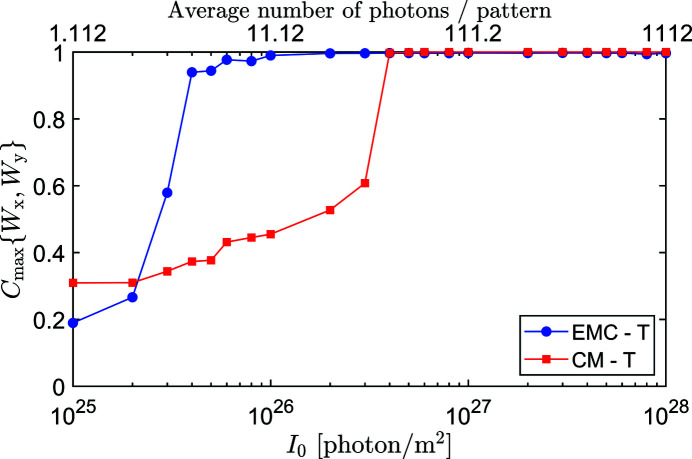
The maximums of Pearson correlation between circular 1D intensity distributions *W*_*x*_ and *W*_*y*_ as rotated relative to each other, for lysozyme at scattering angle 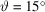 and various incident XFEL fluence values. *W*_*x*_ denotes the intensity distribution reconstructed by either EMC (blue circles) or CM (red squares). *W*_*y*_ refers to the reference intensity distributions *W*_T_ or *W*_0_. *C*_max_ is calculated in all four combinations, but only correlation with *W*_T_ is plotted, the one with *W*_0_ would overlap almost perfectly.

Both the EMC and the CM method were tested for this simple 1D model. Since both the ‘measured’ and the model intensities are periodic 1D functions of the rotation angle φ, there is no need for the compression and expansion steps. After convergence (*i.e.* when no further change was observed between iterations) we compared the resulting intensity distribution *W*(φ_*m*_) with *W*_T_(φ_*m*_) and with the noiseless model intensity distribution *W*_0_(φ_*m*_). The *W* distribution was reconstructed in a random orientation relative to *W*_T_ and *W*_0_. The Pearson correlation (Rodgers & Nicewander, 1988 ▸) was calculated for all possible relative rotations between *W* and *W*_T_ (or *W* and *W*_0_), and the maximum was taken as a measure of the accuracy of the result. These *C*_max_{*W*, *W*_T_} and *C*_max_{*W*, *W*_0_} values are plotted as a function of the incident XFEL fluence *I*_0_ in Fig. 3 ▸. For this simple 1D test case and relatively large *N*_data_, *C*_max_{*W*, *W*_T_} and *C*_max_{*W*, *W*_0_} are not much different. As expected, the more sophisticated EMC method performs better than the CM method. It can solve the orientation problem for an order of magnitude less photons than the simpler CM algorithm. The lowest XFEL intensities *I*_0_ that can be solved by the EMC and CM methods are 4 × 10^25^ and 4 × 10^26^ photons m^−2^, corresponding to an average of 4.46 and 44.6 photons in the ‘measured’ 1D diffraction patterns, respectively. However, when both methods can find the solution, the resulting intensity distribution is slightly more accurate for the CM method. For XFEL fluences *I*_0_ ≥ 2 × 10^27^ photons m^−2^, the CM method gives the ‘true’ distribution *W* = *W*_T_ with all patterns perfectly oriented, while the EMC method still gives some weights to a few slightly misoriented patterns.

### 4.2. Full orientation problem in 3D   

The original orientation problem of SPI is more challenging than the above simplified 1D problem. First of all, the measured patterns are 2D, while the sought intensity distribution is 3D. In the expansion and compression steps, the 3D distribution is interpolated into the 2D grid and *vice versa*. In the EMC method we used linear interpolation, while in the CM method the nearest grid point was selected. In the compression step, pixels of many 2D patterns give contributions to a single voxel of the 3D distribution, leading to a slight smoothing in the reciprocal space.

**Figure 4:**
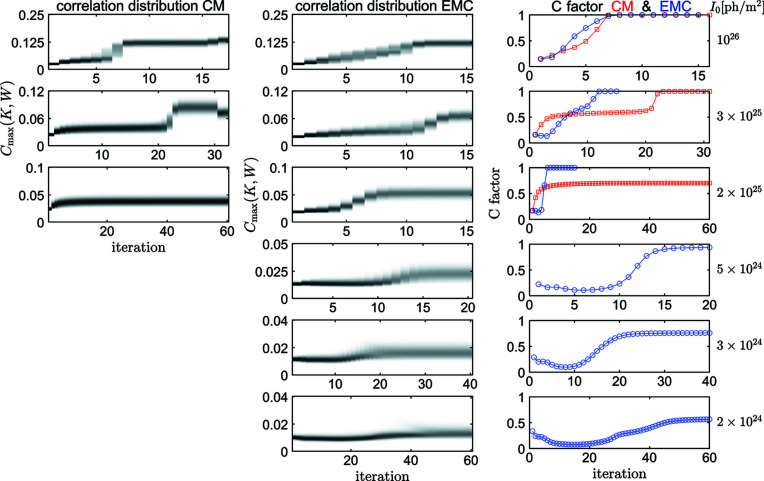
Development of the correlation distributions for the CM (left column) and EMC (middle column) methods at various incident XFEL fluences (indicated at the right-hand side of the figure). The grayscale is linear between zero (white) and the largest value (black). Right column: development of the *C* factor for the CM (red squares) and EMC (blue circles) methods.

We applied the EMC and the CM methods to find the relative orientations of the diffraction patterns of the two test molecules. In all cases, we continued the iteration until there was practically no change in the results. After convergence of the CM algorithm, the orientations of the patterns were further refined (Tegze & Bortel, 2012 ▸). The maximum of the correlation *C*_max_{*K*, *W*} between each pattern and the expanded model slices (*i.e.* the correlation in the best orientation) was calculated for every iteration. This correlation is an essential part of the CM method only (Tegze & Bortel, 2012 ▸), but we calculated it for the EMC method as well. We used the distribution of these correlations to monitor the iteration process. A sudden jump in this correlation distribution indicates finding the solution of the orientation problem. The left two columns of Fig. 4 ▸ show the development of the correlation distribution for both methods at various incident fluences in the case of RNA polymerase II.

Several methods have been suggested to validate the results of the orientation process. Fourier shell correlation (FSC; Harauz & van Heel, 1986 ▸), developed for cryo-EM, is frequently used to validate the reconstructed 3D intensity distribution, and to estimate the spatial resolution for SPI data as well (Nakano *et al.*, 2018 ▸; Rose *et al.*, 2018 ▸; von Ardenne *et al.*, 2018 ▸; Ayyer *et al.*, 2019 ▸; Giewekemeyer *et al.*, 2019 ▸; Poudyal *et al.*, 2020 ▸). This and some other methods (Yoon *et al.*, 2016 ▸; Liu *et al.*, 2018 ▸) rely on dividing the measured dataset into two or more parts and compare the independently recovered intensity distributions. However, Shen *et al.* (2021 ▸) have shown that these methods suffer from serious problems. Most notably, the correlation can grow with increasing orientation disorder when approaching the powder average. They suggested a validation method based on information theory, which is free of these problems. However, calculations of the suggested measures of orientation uncertainties are rather complicated and not practical for orientation methods other than EMC, where most of the probability distributions necessary are already computed. In one of our earlier articles (Tegze & Bortel, 2016 ▸) we introduced correlation maps (distributions in orientation space of correlations between a diffraction pattern and slices of the 3D intensity volume) and the *C* factor, a single figure of merit to indicate the progress and convergence of the orientation algorithm. The *C* factor is defined as the ratio of the diffraction patterns with peaks well above the noise level in the correlation maps. The developments of the *C* factor at various XFEL fluences for both EMC and CM is shown in the right column of Fig. 4 ▸.

**Figure 5:**
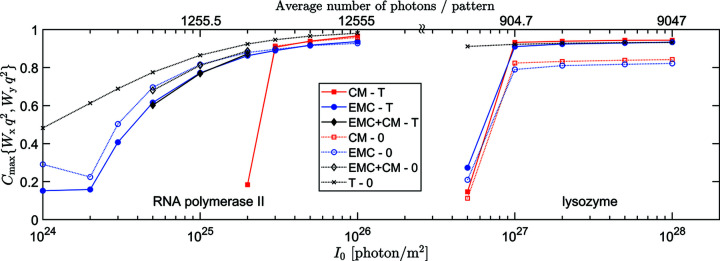
The maximums of Pearson correlation between intensity volumes *W*_*x*_ and *W*_*y*_ as rotated relative to each other, for lysozyme (right) and RNA polymerase II (left) at various incident XFEL fluence values. The upper scale (number of photons per pattern) is different for the two molecules. *W*_*x*_ denotes the intensity distribution reconstructed by either EMC (blue circles), CM (red squares), a combination of them (black diamonds, see the main text for details) or using the true orientations (black crosses). *W*_*y*_ refers to the reference intensity distributions *W*_T_ (full symbols and solid lines) or *W*_0_ (empty symbols and dotted lines).

We can test the accuracy of the reconstructed 3D intensity volume *W* by comparing with reference volumes produced in the simulation step. We use two reference volumes: *W*_0_, the ideal noiseless 3D model intensity distribution calculated directly from the known atomic structure, and *W*_T_, constructed from the simulated noisy patterns using their true orientations. Since the shapes of the patterns used for the reconstruction are spherical caps in the reciprocal space with the origin at their center, the shapes of the reconstructed volumes *W* and *W*_T_ are spherical. The 3D intensity distribution *W* is recovered in a random orientation. Therefore, before comparison we have to rotate *W* to the best-fitting orientation. We first rotate *W* with all possible Euler angles on a 3D orientation grid, interpolate to the 3D grid in reciprocal space and find the angles with maximum Pearson correlation with *W*_0_ or *W*_T_. Then we refine the three Euler angles by a nonlinear maximization method. Comparison of *W* with *W*_T_ gives information on the errors due to the misorientation of the patterns. In the ideal case, when the true orientations are found, the correlation would be near to unity (the small deviation is due to interpolation errors). On the other hand, comparing with *W*_0_ shows the effects of all errors (misorientation and Poisson noise) and the correlation approaches unity only when the Poisson noise is negligible (the XFEL pulse fluence is very high). The scattered intensity decreases approximately with the inverse square of the length of the scattering vector **q**, therefore we applied *q* ^2^ weighting to both *W* and *W*_T_ (or *W*_0_) before calculating the correlation. The resulting *C*_max_{*Wq* ^2^, *W*_T_ *q* ^2^} and *C*_max_{*Wq* ^2^, *W*_0_ *q* ^2^} correlations are presented in Fig. 5 ▸. In principle, the correlation to *W*_T_ would show how well the patterns are oriented. However, small errors in the orientation and interpolation errors and averaging in the compression step lead to differences in the noise in *W* and *W*_T_, and a considerable decrease in the correlation. Since the model intensity *W*_0_ is without noise, for the more complex RNA polymerase II molecule at low XFEL pulse fluences, the correlation to *W*_0_ (dotted lines in Fig. 5 ▸) can be higher than the correlation to *W*_T_ (solid lines). For reference, we also plotted the correlation between *W*_T_ and *W*_0_ in Fig. 5 ▸ (black cross symbols and dotted lines).

Surprisingly, for the relatively small lysozyme, the results of the two methods do not differ much. Both methods are successful for XFEL fluences *I*_0_ ≥ 10^27^ photons m^−2^, corresponding to an average of 905.5 photons in a pattern. As in our 1D tests, the CM method gives slightly more accurate results. The increase in accuracy is partly due to the refinement of the orientations in the final step of the CM method. Unfortunately, the same refinement is not possible for the EMC method, where not only the best, but all orientations are used to construct the 3D intensity distribution.

For the more realistic case of RNA polymerase II, we found that the CM algorithm can solve the orientation problem for pulse fluences *I*_0_ ≥ 3 × 10^25^ photons m^−2^, corresponding to an average of 927.4 photons in a pattern. This incident X-ray pulse fluence is near to the limit of the capabilities of present XFEL sources. The EMC method provides again slightly less accurate results for the same fluence region, but can also give solutions for even lower fluences (*I*_0_ ≥ 5 × 10^24^ photons m^−2^, an average of 156.1 photons per pattern). However, the accuracy of the solution strongly decreases with decreasing X-ray fluence (blue circles in Fig. 5 ▸).

We tried to improve the results of the EMC method by applying a few extra iterations by the CM algorithm. We hoped that in the incident X-ray fluence region where the CM method by itself failed to converge, replacing the probability distribution in EMC by a Dirac delta function and then refining the orientation angles would give better results. However, this combination of the two methods produced only marginal improvement in the correlation with *W*_T_ or *W*_0_ at *I*_0_ = 2 × 10^25^ and even a slightly worse correlation at *I*_0_ = 5 × 10^24^ (black diamonds in Fig. 5 ▸).

**Figure 6:**
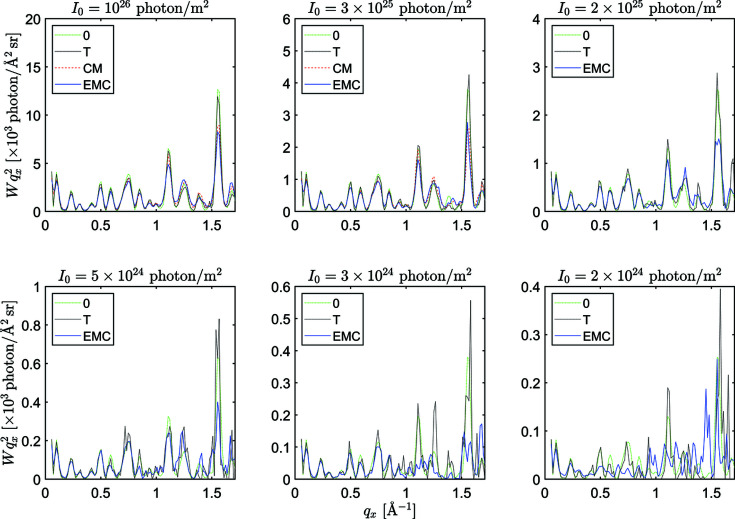
Intensity *W* reconstructed by the EMC (blue solid line) method, the CM (red dashed line) method, and using the true orientations (*W*_T_, black dotted line) for RNA polymerase II along a radial direction at various incident XFEL fluences *I*_0_ (indicated at the top of the panels). The shape of the noiseless model intensity (*W*_0_, green dash-dotted line) is independent of *I*_0_.

We plotted the reconstructed intensity *W* along a radial direction in *q* space in Fig. 6 ▸ at six selected *I*_0_ values. The results of the two algorithms (EMC: blue solid lines: CM: red dashed lines) are compared with the reconstruction using the true orientations (*W*_T_, black dotted lines) and the noiseless model intensity (*W*_0_, green dash-dotted lines). At high XFEL pulse fluences, *I*_0_ > 2 × 10^25^, the agreement between all four curves is quite good in the full *q* region. For lower *I*_0_ values the CM method does not converge to a meaningful solution. As *I*_0_ decreases, the reconstruction by EMC (and to a lesser extent, even *W*_T_) deviates more from the model intensity *W*_0_ in the high-*q* region.

**Figure 7:**
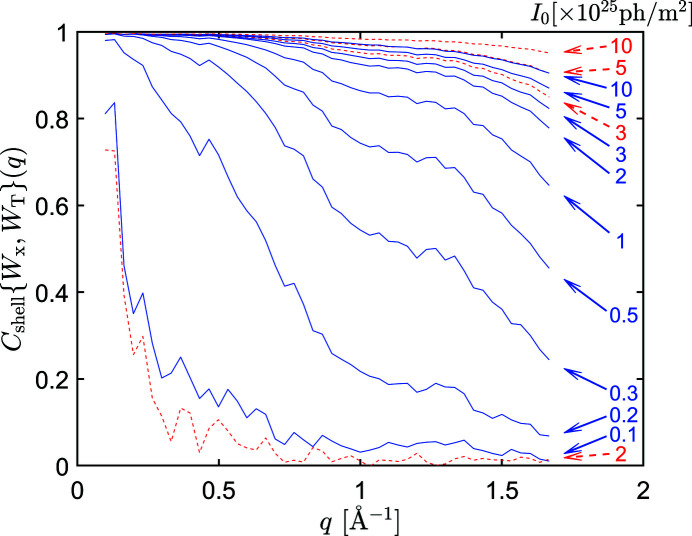
FSC between the 3D intensity volume *W* reconstructed by the EMC (blue solid lines) and CM (red dashed lines) methods and the ideally reconstructed intensity *W*_T_ at various incident XFEL fluences for RNA polymerase II.

The loss of accuracy with decreasing incident X-ray fluence is not uniform within the recovered intensity sphere. Misorientation of the patterns and relative noise increasing with *q* both lead to higher inaccuracies at large *q* values, thus decreasing resolution. We can investigate the loss off resolution by calculating the FSC between the 3D intensity volume *W* reconstructed by either method and the ideally reconstructed intensity *W*_T_ or the noiseless 3D model intensity distribution *W*_0_. We noted earlier that FSC, calculated between volumes reconstructed from two independent sets of measured data, suffers serious problems when used for characterization of the resolution errors (Shen *et al.*, 2021 ▸). However, when FSC is calculated between a measured intensity volume *W* and model intensities *W*_0_ with no errors or *W*_T_ with well known errors (Poisson noise and interpolation errors), most of these problems disappear. In Fig. 7 ▸ we show the FSC between *W* and *W*_T_ for both methods at various XFEL pulse fluences. One can see that the loss of accuracy becomes significant at higher-resolution shells as the XFEL pulse fluence decreases.

**Figure 8:**
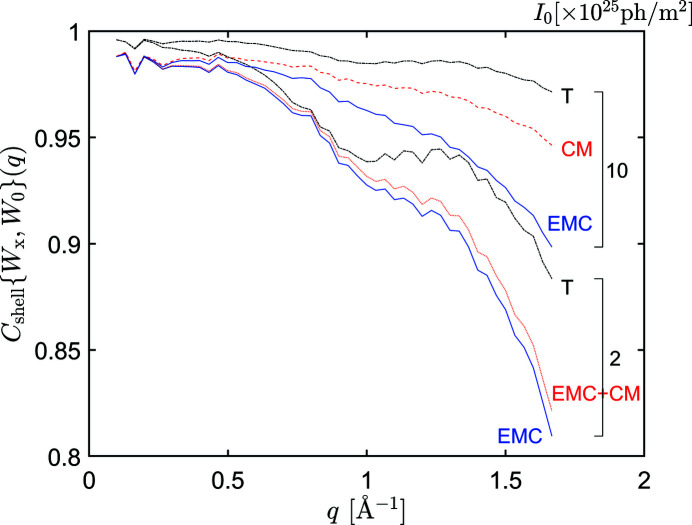
FSC between the 3D intensity volume *W* reconstructed by the EMC (blue solid lines) method, the CM (red dashed lines) method or a combination of both methods (red dotted line), and using the true orientations (*W*_T_, black dash-dotted line) and the noiseless model intensity *W*_0_, at *I*_0_ = 2 × 10^25^ and 10^26^ for RNA polymerase II.

In Fig. 8 ▸, we plotted the FSC between the reconstructed intensities and the noiseless model intensity*W*_0_ for two values of the XFEL pulse fluence *I*_0_. At *I*_0_ = 10^26^, the FSC for the CM method (red dashed line) is much nearer to the FSC for the ideally reconstructed intensity *W*_T_ (black dash-dotted line) than the FSC for the EMC method (blue solid line). This indicates again that the CM method, when it can solve the orientation problem, gives more accurate results than the EMC method. At the lower XFEL pulse fluence *I*_0_ = 2 × 10^25^, the CM algorithm did not converge. Instead, we plotted here the FSC for the results of the combined EMC + CM method (a few iterations of CM after convergence by EMC, red dotted line in Fig. 8 ▸). In this case, the improvement over the results of the EMC method (blue solid line) is small.

**Figure 9:**
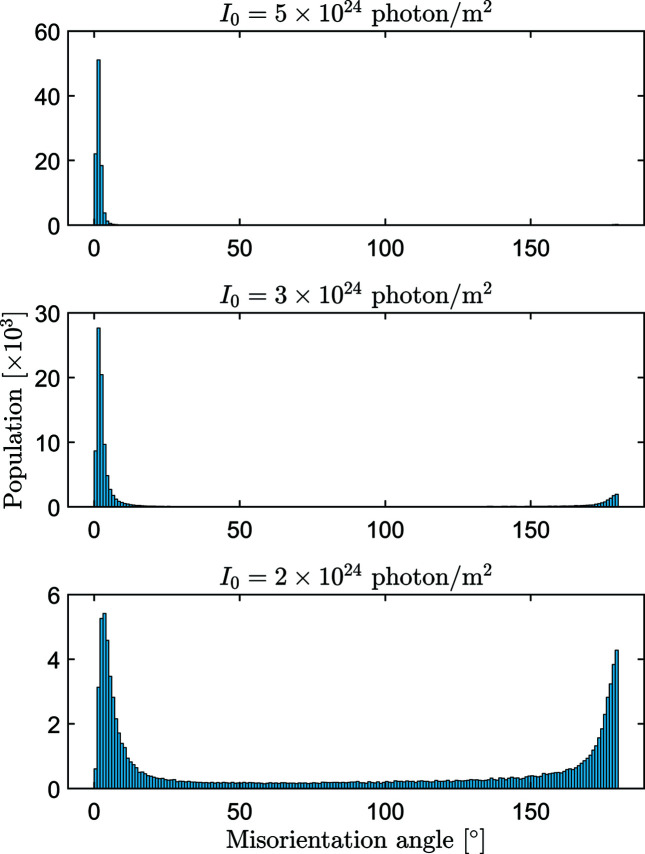
Distribution of the misorientation angle for the converged results of the EMC method at three different XFEL pulse fluences (indicated at the top of the panels) for RNA polymerase II.

The origin of the accuracy loss at high *q* values is twofold. First, the signal-to-noise ratio of the patterns and thus that of the reconstructed 3D intensity volume decreases with decreasing *I*_0_, even for the case where the patterns are perfectly oriented (see the black cross symbols and dotted lines in Fig. 5 ▸). As the number of scattered photons decreases with the scattering angle and the number of patterns contributing to a voxel also decreases, the accuracy strongly decreases with *q* (see the black dash-dotted lines in Fig. 8 ▸). The second reason for the loss of accuracy is the imperfect orientation of the patterns. The CM method, when the signal-to-noise ratio is not sufficient to orient a pattern, simply fails to converge to a meaningful solution. The EMC method behaves differently at lower XFEL pulse fluences. It converges to a solution but the result deviates from the ideal solution *W*_T_ for lower XFEL pulse fluences (see the increasing difference between the blue circles and the black cross symbols in Fig. 5 ▸). Since from the simulation we know the true orientations of the patterns, we can calculate the angular error of the orientation of each pattern. First, we find the best orientation (the one with the highest correlation to the reconstructed 3D volume *W*) of a pattern, then we calculate the misorientation angle, *i.e.* the smallest angle of rotation to bring the pattern to its true orientation. In the calculation we take into account the rotation necessary to reach the highest correlation between *W* and *W*_T_. The distribution of the misorientation angles is shown in Fig. 9 ▸ for XFEL pulse fluences between 2 and 5 × 10^24^ photons m^−2^. At *I*_0_ = 5 × 10^24^, the misorientation angles are small, typically a few degrees. As *I*_0_ decreases, the misorientation peak widens and a second peak appears at 180°, which is the largest possible misorientation. At *I*_0_ = 2 × 10^24^ the second peak becomes as large as the first one. This means that the algorithm cannot distinguish between two orientations of a pattern separated by a 180° rotation in orientation space. It is easy to understand why this happens if we consider the Friedel symmetry of elastic X-ray scattering. According to Friedel’s law, in the absence of resonant scattering, the scattered intensity is centrosymmetric in the reciprocal space. If the measured patterns were flat, then they would be centrosymmetric as well. As an inversion is identical to a twofold rotation in 2D, the centrosymmetry introduced by Friedel’s law would appear as an exact 180° rotation ambiguity in this hypothetic planar pattern orientation problem. However, in SPI the patterns in the reciprocal space are not flat, but spherical caps. Near the center at *q* = 0, they are nearly centrosymmetric, but as the scattering angle increases, the deviation from centrosymmetry becomes larger. This deviation makes it possible for the orientation methods to distinguish between the two orientations of a pattern related by a 180° rotation about its center. When this deviation from centrosymmetry disappears in the noise (the relative noise is largest at the edge of the pattern), then the orientation methods place the pattern in both orientations with ∼50% probability. At low *q* values this does not cause large errors but at higher *q* values the accuracy of the reconstructed intensity is strongly degraded.

**Figure 10:**
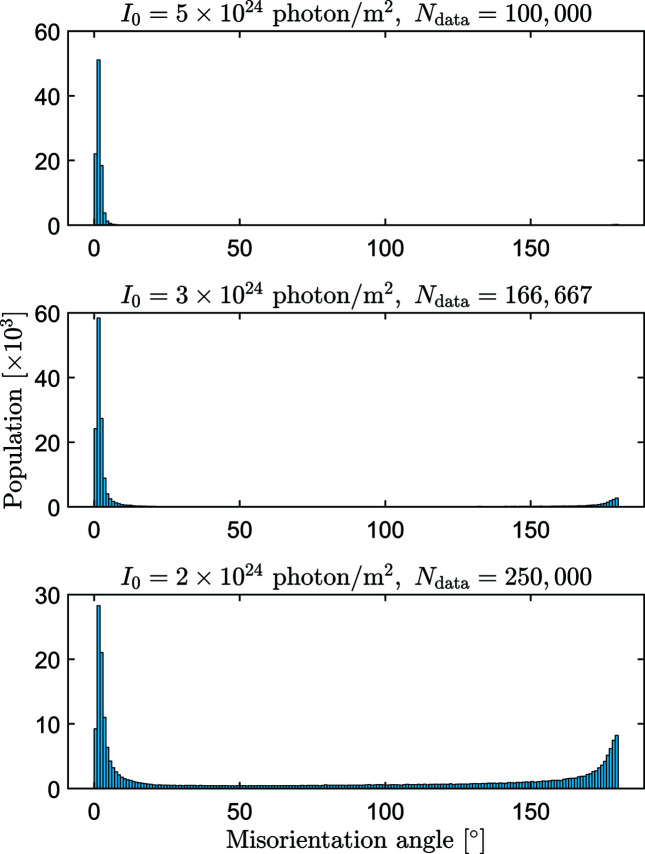
Distribution of the misorientation angle for the same pulse fluences as in Fig. 9 ▸, but using different numbers of diffraction patterns. The numbers of patterns *N*_data_ (indicated at the top of the panels) are chosen to keep the total number of collected photons constant. The histogram in the top panel is identical to that of Fig. 9 ▸, we included it here for easier comparison.

We also calculated the misorientation angles for the same pulse fluences as in Fig. 9 ▸, but using different numbers of diffraction patterns (Fig. 10 ▸). The numbers of patterns *N*_data_ are chosen to keep the total number of collected photons constant. Increasing *N*_data_ decreases the noise of the assembled 3D intensity distribution. From comparison of the two figures, it is clear that the increased number of patterns can only partly compensate the decreasing number of photons in the individual patterns while keeping the total number of collected photons constant.

## 5. Phase retrieval   

The 3D intensity distribution reconstructed by the orientation algorithm is the input for the next phase-recovery step in the evaluation process. The accuracy of the intensity distribution affects the quality of the resulting electron density. Moreover, errors in the 3D intensity distribution may prohibit the recovery of the phases and producing an electron density at all. The ideal intensity distribution is the squared modulus of the Fourier transform of the electron density. The electron density is always real and non-negative. This places serious constraints on the intensity distribution, which are usually not satisfied if it contains errors. The reality of the electron density requires the intensity to be centrosymmetric by Friedel’s law. This can be easily satisfied by replacing the intensity *W*(**q**) with its symmetric average [*W*(*q*) + *W*(−*q*)]/2. Unfortunately, there is no simple rule for the intensity distribution to ensure the non-negativity of the electron density. There are several iterative algorithms (Fienup, 1982 ▸; Bauschke *et al.*, 2004 ▸; Luke, 2005 ▸; Oszlányi & Sütő, 2008 ▸) to find a set of phases which approximately satisfy this condition. However, these algorithms may fail, if the errors in the reconstructed 3D intensity distribution are too large.

**Figure 11:**
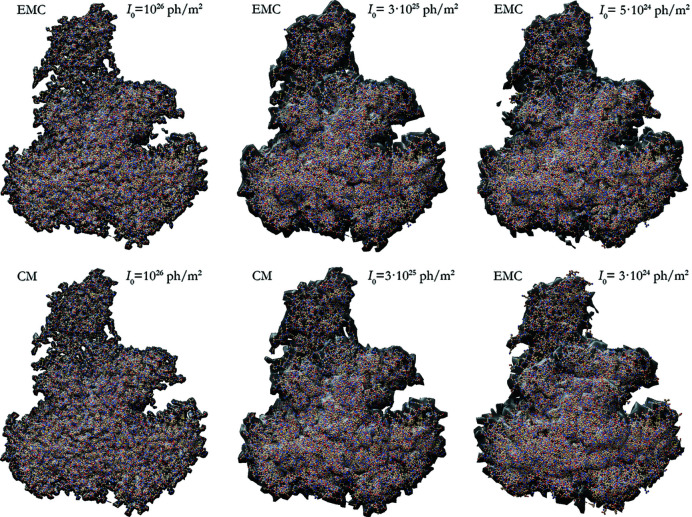
Reconstructed electron density (gray surface) of RNA polymerase II for various XFEL pulse fluences (indicated on the panels). The orientation problem was solved by the CM (bottom left and middle panel) and EMC methods (all other panels). All reconstructed electron densities were rotated and shifted to achieve the best overlap with the original structure (ball-and-stick model) using the *Chimera* program (Pettersen *et al.*, 2004 ▸).

We used Fienup’s hybrid input–output (HIO) algorithm (Fienup, 1982 ▸) with parameter β = 0.7 in conjunction with a shrink-wrap constraint (Marchesini *et al.*, 2003 ▸) to recover the electron density of RNA polymerase II. The threshold for the shrink-wrap constraint was 5% of the maximum value of the electron density. The unmeasured regions in the corners of the superscribed cube of the reconstructed intensity volume and in the center were set to zero at the start and allowed to have any value at later iterations. The initial phases were random numbers between 0 and 2π. After convergence of the HIO algorithm, 50 iterations of the error-reduction algorithm (Fienup, 1982 ▸) were executed. In Fig. 11 ▸ the reconstructed electron densities for various XFEL pulse fluences *I*_0_ are compared with the atomic model of the molecule. For 3 × 10^25^ ≥ *I*_0_ ≥ 5 × 10^24^ it was necessary to reduce the size of the intensity volume by a factor of 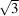 to the inscribed cube in order to find a solution of the phase problem. At *I*_0_ = 3 × 10^24^ a reduction by a factor of two (one eighth of the original cube volume) was needed. Below this value we were unable to find the correct phases, even when the size of the *q* region was further reduced. At higher XFEL pulse fluences there is no observable difference between the results of the EMC and CM orientation methods. At lower X-ray fluences (where only the EMC algorithm converged) the resolution and the accuracy of the reconstructed electron density visibly decrease.

## 6. Conclusions   

We tested the efficiency of two methods, EMC and CM, for orienting the noisy SPI patterns and reconstructing a consistent 3D intensity distribution. The EMC algorithm was slightly modified to increase its computing speed. We found that at higher XFEL pulse fluences both the EMC and the CM algorithms give reliable results. However, the 3D intensity volume reconstructed by the CM method is more accurate than the one by the EMC method. The reason for this is the following: the EMC method uses the probability distributions as weights for the different orientations (defined on a grid) of a measured pattern. These distributions become narrow when convergence is reached at high XFEL pulse fluences. However, due to oversampling of the reciprocal space, this narrow width can still lead to some smearing of the resulting intensity distribution. In contrast, the CM method can find the single best orientation of a measured pattern and can even refine its orientation angles.

For lower incident XFEL fluences, the CM method fails, while the more sophisticated EMC method can still converge to a meaningful solution. However, with decreasing XFEL pulse fluence the accuracy of the resulting 3D intensity distribution quickly deteriorates. We found that applying the CM method to the results of the EMC method can only marginally improve them. The decrease in accuracy is most pronounced at the high-*q* part of the results and leads to a loss of resolution in the reconstructed electron density. This decrease in accuracy is because the algorithm cannot distinguish between two orientations of a pattern (related by a 180° rotation) when the signal-to-noise ratio is low.

While in this simulation it was easy to verify the results, we would like to stress the importance of using reliable figures of merit (*e.g.* the *C* factor; Tegze & Bortel, 2016 ▸) and correlation maps (Tegze & Bortel, 2016 ▸, 2018 ▸) to validate the results in the case of real measurements.

## Figures and Tables

**Table 1:** Parameters of the test molecules, the simulations and the orientation processes

Name of the molecule	Lysozyme	RNA polymerase II
Weight (kDa)	14	509
Largest diameter *D* (Å)	51	167
X-ray wavelength λ (Å)	1	1.91
Range of scattering angle ϑ_min_–ϑ_max_ (°)	1–30	1–30
Pattern pixel size (polar × azimuthal), Δϑ × Δφ (°)	1 × 2	0.25 × 0.5
Resolution *d* (Å)	1.9	3.7
Complexity *R* = *D*/*d*	26	45
Number of patterns *N*_data_	20 000	100 000
Number of grid points in the (*Φ*, *Θ*) orientation subspace *N*_R_	5292	5292
Number of orientations *N*_R_ *N_φ_*	952 560	3 810 240
Number of grid points (voxels) in the reciprocal space	61^3^ = 226 981	239^3^ = 13 651 919
Computation time per iteration (CM/EMC) (s)	18/47	311/746

## Appendix A Modifications of the EMC algorithm to improve computational efficiency

We use the notations of Loh & Elser (2009 ▸) when possible and introduce new notations only where necessary.

The 2D detectors used for collecting the scattered photons in a SPI experiment are usually flat with rectangular pixels and (after some calibration and processing) provide photon counts *K_ik_* for frame *k* and pixel *i*. As a first step, we divide the photon counts by the solid angles of the individual pixels and correct with the polarization factor. The resulting intensities are interpolated to a regular (*ϑ_ℓ_*, φ_*m*_) polar grid on the surface of the Ewald sphere. The polar angle  corresponds to the scattering angle (conventionally 2θ in crystallography) and the azimuthal angle   corresponds to a rotation about the incident X-ray beam, and together they define the scattering vector:

where *k* is the wavenumber.

The step Δϑ is chosen to approximately match the detector pixel size, and Δφ = 2π/*N*_φ_ ≃ Δϑ/sin ϑ_max_, where ϑ_max_ = *N*_ϑ_Δϑ is the scattering angle at the edge of the detector. Finally, we multiply the intensities by the solid angles  to get ‘photon counts’ again. The transition from detector pixels to polar grid introduces some smoothing of the data due to the interpolation used. The resulting *K_ℓmk_* are not really photon counts, as they are not integers, and the Poisson statistics are not strictly valid any more. However, all expressions derived from the Poisson distribution can be extended to non-integers (formally by replacing factorials with gamma functions) without adverse effects on the performance of the EMC method.

In the E step of the EMC algorithm, the 3D model *W*[**q**] is expanded to the same (*ϑ_ℓ_*, *φ_m_*) polar grids on the surface of the Ewald sphere that has been rotated by the **R** *_j_* rotation operators. The rotation operation can be described as subsequent rotations about axes *z*, *x* and *z* by Euler angles Ψ, *Θ* and *Φ*, respectively. Since the first rotation by Ψ is equivalent to a rotation of the polar grid about the *z* axis (the direction of the incident X-ray beam), we can handle this rotation in a different way. The other two rotations by (*Θ*, *Φ*) can be represented as spherical coordinates of points on the unit sphere. We set up an approximately uniform grid on the surface of the sphere [for details, see Tegze & Bortel (2012 ▸)] with grid points (*Θ_r_*, *Φ_r_*) and corresponding rotation operators **R** *_r_*. Using this notation, *W_ij_* in the original EMC algorithm becomes *W_ℓ,m,r,m′_*. Here the indices *ℓ, m* refer to the points of the polar grid, and the indices *r, m′* refer to rotations **R** *_r_* and **R** *_m′_* by (*Θ_r_*, *Φ_r_*) and *Ψ_m′_*, respectively. If we choose the same grid for Ψ as for φ, then the rotation **R** *_m′_* is the same as a shift in the index *m*, and *W_ℓ,m,r,m′_* = *W_ℓ,m+m′,r_*. To summarize the changes in the notation:

The most time-consuming step of the EMC algorithm is the computation of the probabilities *P_jk_* (*W*). The probabilities *P_jk_* (*W*) are the normalized versions of the likelihood functions *R_jk_* (*W*) [see equation (9) of Loh & Elser (2009 ▸)]. Following equation (18) of Loh & Elser (2009 ▸), the logarithm of the likelihood function can be written as

Here we used the property that the sum for all pixels of *W_ℓ,m+m′,r_* is independent of *m′*. Now we can apply the cross-correlation theorem (Weisstein, 2021 ▸) for the first sum over *m* on the right-hand side of equation (3):

Here FFT refers to the fast Fourier transform along index *m* and the asterisk denotes the complex conjugate. Thus the logarithm of the likelihood function becomes

The complexity of calculating the sum for each *m′* is *O*(*N_φ_* ^2^), while the same for the Fourier transform is *O*(*N_φ_* log *N_φ_*). This results in a speedup of the calculation of the order of *N_φ_*/(3 log *N_φ_*). Since Δφ is usually ∼1° and  , we estimate that the gain in speed is somewhere between 10 and 100.

We can apply an equivalent transformation in the compression step. We separate again the rotations *Ψ_m_* and (*Θ_r_*, *Φ_r_*), and calculate a partially compressed model by averaging for the index *m′*:

Here  are the probabilities normalized for all measurements [see equation (11) of Loh & Elser (2009) ▸]. We use again the cross-correlation theorem:

Now we map the  values to the 3D grid [as in equation (14) of Loh & Elser (2009 ▸)]:

where **p** denotes a spatial-frequency sampling point in the 3D intensity space and *f*(**q**) represents the interpolation weights.
